# The ubiquitin-proteasome system in Alzheimer’s disease: mechanism of action and current status of treatment

**DOI:** 10.3389/fnagi.2025.1730206

**Published:** 2025-12-03

**Authors:** Shanshan Jia, Quan Li, Xue Rui, Wen Qin, Wenhui Zhang, Jinjin Dou, Xiwu Zhang

**Affiliations:** 1 The First Clinical Medical College of Heilongjiang University of Chinese Medicine, Harbin, China; 2 Basic Medical College of Heilongjiang University of Chinese Medicine, Harbin, China; 3 The Second Clinical Medical College of Heilongjiang University of Chinese Medicine, Harbin, China; 4 Department of Cardiovascular, The Fourth Hospital of Heilongjiang University of Traditional Chinese Medicine, Harbin, China; 5 Experimental Training Center of Heilongjiang University of Chinese Medicine, Harbin, China

**Keywords:** Alzheimer’s disease, ubiquitination, ubiquitin-proteasome system, Aβ protein, tau protein

## Abstract

Alzheimer’s disease (AD) is one of the most common neurodegenerative disorders; current therapies can neither cure AD nor prevent its progression. The pathological hallmark of AD is the excessive deposition of abnormal proteins in the brain, primarily including β-amyloid (Aβ) and phosphorylated Tau proteins. The ubiquitin-proteasome system (UPS), a central intracellular protein degradation mechanism that removes misfolded proteins and maintains cellular homeostasis by inhibiting aberrant protein aggregation, plays an important role in the regulation of various physiological functions, as well as in the development of disease. Any abnormality in this process leads to protein misfolding and aggregation, and the accumulation and aggregation of ubiquitinated proteins is a common feature of many neurodegenerative diseases, including AD. A growing number of studies have confirmed the significance of UPS in the AD process, which may act in conjunction with other mechanisms leading to the development of AD, and may even be the direct cause of AD. UPS offers a whole new possibility for the development of drugs for AD prevention and treatment, as well as new strategies and approaches for the treatment of neurodegenerative diseases. Therefore, this review is based on UPS, describes the possible mechanisms of action of UPS in AD, and summarizes the preclinical studies of modulating UPS for the treatment of AD.

## 1 Introduction

AD is a degenerative disease of the central nervous system characterized by progressive cognitive dysfunction and behavioral decline (Scheltens et al., 2021). The main pathological features of AD are senile plaques (SPs) formed by excessive deposition of Aβ in the brain and neurofibrillary tangles (NFTs) formed by over phosphorylation of Tau proteins, as well as loss of neurons and alteration of synaptic number and function (Blennow and Zetterberg, 2018). The pathogenesis of AD has not been clearly elucidated, and the current mainstream theories include the amyloid cascade hypothesis, the Tau protein phosphorylation hypothesis, the cholinergic hypothesis, the mitochondrial cascade hypothesis, etc. (Tiwari et al., 2019; Ju and Tam, 2022; Ashleigh et al., 2023). Currently, there are about 55 million AD patients worldwide, and with the accelerated global aging process, the prevalence of AD is climbing year by year. And it is expected that in 2050, this number may increase by two times (Janoutová et al., 2021). According to global reports, the estimated cost of treating and caring for AD patients is about $305 billion by 2020, and this cost is expected to exceed $1 trillion by 2050 (Wong, 2020).

There are two sets of protein degradation pathways in eukaryotes, one is the UPS and the other is the autophagy lysosomal pathway (ALP) (Watanabe et al., 2020). ALP is a lysosomal degradation pathway, which mainly works to degrade macromolecular proteins (Zeng et al., 2019). UPS is a major non-lysosomal degradation pathway that maintains protein homeostasis in cells of the nervous system, degrades 80 ~ 90% of misfolded, damaged, or mutated proteins in eukaryotic cells, and plays an important role in the regulation of the cell cycle, apoptosis, and DNA transcription and repair processes (Dagar et al., 2023). UPS dysfunction is affected by a variety of factors, of which age is one of the major ones (Thellung et al., 2022). Abnormalities in any of the links of the UPS pathway can lead to a decrease in its degradation of proteins, which can, in turn lead to aberrant aggregation of proteins, disrupting cell function and even leading to cell degeneration and death. Perry et al. (1987) first found a large amount of ubiquitin in the brains of AD patients. It has also been found that proteasome activity in the hippocampus, Para hippocampus, middle temporal gyrus, and inferior parietal lobule of the brain of AD patients is significantly reduced (Keller et al., 2000). The dysfunction of UPS leads to a decrease in the clearance of abnormal proteins from the brain of AD patients, exacerbating the accumulation of neurotoxic proteins, which in turn disrupts protein homeostasis and induces cellular malfunction. Conversely, these excessively deposited neurotoxic proteins further inhibit the functionality of the UPS, thus promoting the further development of AD (Liu et al., 2021).

In this review, we mainly summarize the functions and roles of the major components of the ubiquitination process and how ubiquitination affects the homeostasis of the protein environment associated with AD, as well as sort out the current preclinical studies. Through the review of the above ingredients, we hope to provide some useful help for the drug development of UPS ingredients.

## 2 The molecular process of UPS

Misfolding and aggregation of proteins is a common feature of several neurodegenerative diseases, and UPS has the role of degrading abnormal proteins and facilitating protein turnover (Gupta et al., 2021). UPS consists of ubiquitin (Ub) and related enzymes, including ubiquitin-activating enzymes (E1s), ubiquitin-conjugating enzymes (E2s), ubiquitin ligases (E3s), 26S proteasomes, targeting proteins, and deubiquitinating enzymes (DUBs) (Spano and Catara, 2023).

### 2.1 Ubiquitination process

The process of ubiquitination is an enzymatic reaction involving multiple enzymes. The process begins with the activation of ubiquitin molecule by E1 enzyme under the condition of adenosine triphosphate (ATP) energy supply (Lambert-Smith et al., 2020); Then ubiquitin was transferred to E2 enzyme (Lee et al., 2025); Finally, E3 enzyme (the decisive factor in this process) specifically recognizes the substrate protein and catalyzes the covalent connection of ubiquitin molecules to the substrate protein (Wang D. et al., 2017). The repeated ubiquitination process forms a ubiquitin chain, which is a signal that proteins can be degraded.

### 2.2 Proteasome and degradation

**Figure 1:**
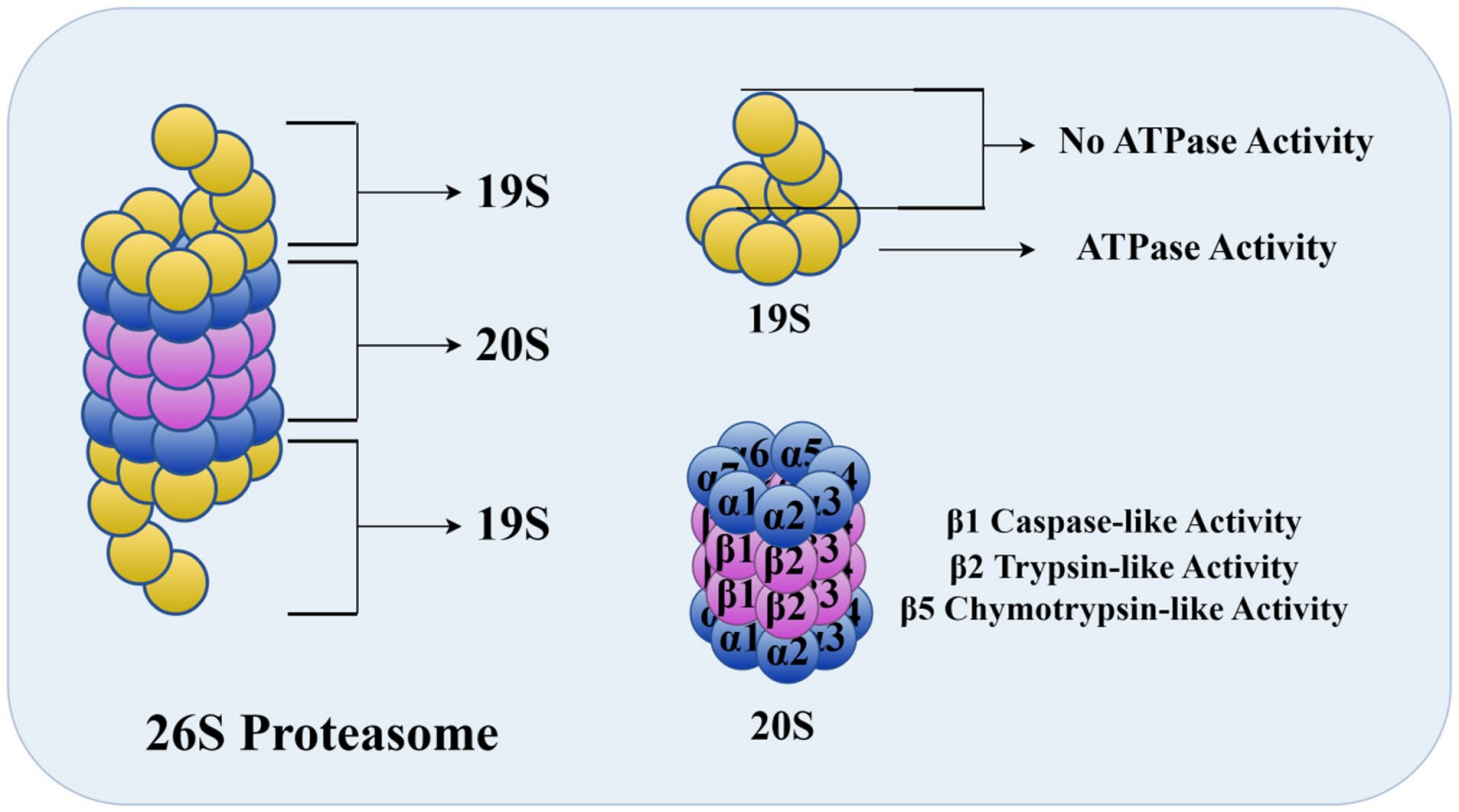
26S proteasome (As shown in the figure, the 26S proteasome consists of two parts, the core particle 20S and the regulatory particle 19S. 19S is divided into a base and a cap; the base part contains ATPase, which is responsible for the recognition of ubiquitin tags and their transfer to the core particle, and the cap part is involved in the recognition and removal of the ubiquitin chain. 20S consists of four stacked rings, and in mammals, there are three β-rings that play active sites for protein hydrolysis, namely β1, β2, and β5. The figure was drawn using Figdraw).

The substrate protein labeled by a polyubiquitin chain was recognized and degraded by the 26S proteasome. The 26S proteasome is the largest and most complex member of the ATP-dependent protease superfamily (Figure 1). Its degradation function can not only remove abnormal proteins, but also control numerous important cellular processes, including cell cycle, DNA replication, transcription, signaling, and stress response (Davidson and Pickering, 2023). After degradation, ubiquitin molecules are released, and free ubiquitin molecules continue to complete the new ubiquitination process.

### 2.3 The process of deubiquitination

**Figure 2:**
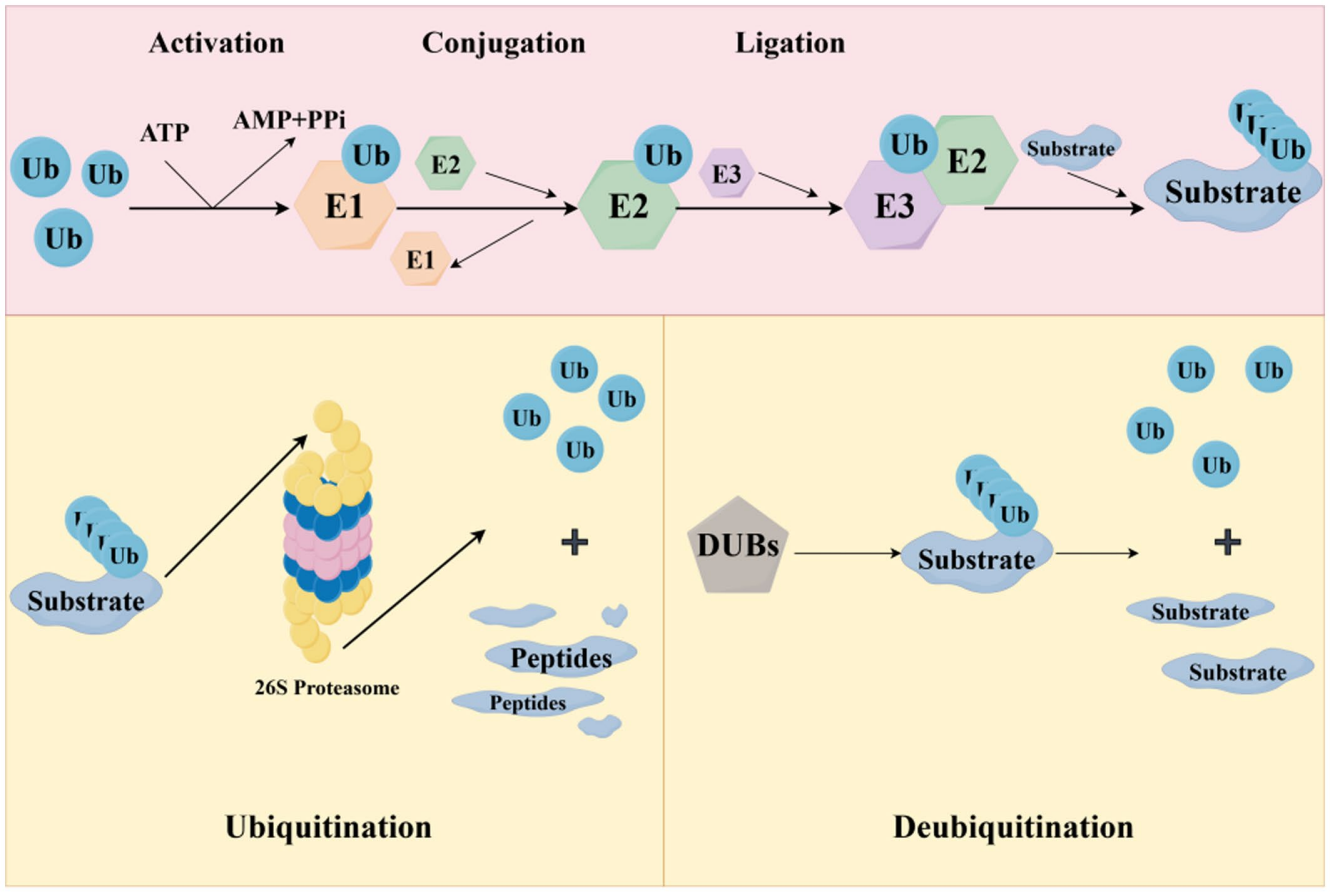
Ubiquitination degradation process and deubiquitination process (The ubiquitination process is realized through a cascade reaction of three enzymes. With ATP supplying energy, the cysteine residue of E1 binds to the glycine residue at the C-terminus of Ub to form a high-energy thioester bond, which in turn activates Ub. The activated ubiquitin molecule is transported to E2 and connects with the cysteine residue of E2 to form a thioester bond. Finally, the activated ubiquitin molecules are transferred to the proteins to be degraded by the specific recognition of E3, and so on. After binding a certain number of ubiquitin molecules, a polyubiquitin chain is formed, and target proteins carrying the polyubiquitin chain are transported to the 26S proteasome for degradation, and ubiquitin molecules are released for reuse in the process. And deubiquitinating enzymes can reverse this process. The figure was drawn using Figdraw).

Ubiquitination modification is not an immutable process; it can be reversed, and the reversible process corresponding to ubiquitination is deubiquitination. This process is driven by deubiquitinases (DUBs) (Figure 2). DUBs can decompose the binding between substrate proteins and ubiquitin molecules, and the dissociated ubiquitin molecules can be reused in the ubiquitination pathway (Xian et al., 2024). About 100 DUBs are expressed in humans. According to the structure and catalytic mechanism of their catalytic domains, deubiquitinases can be divided into five categories: ubiquitin-specific proteases (USPs), ubiquitin C-terminal hydrolases (UCHs), Machado Joseph disease protein domain proteases (MJDs), ovarian tumor proteases (OTUs), and jabl/MPN domain-related metalloisopeptidases (JAMM) (Komander, 2009). Deubiquitinases are involved in many important biological processes, such as cell growth and tumorigenesis, differentiation and development, as well as memory loss and the development of neurodegenerative diseases (Liu et al., 2025).

## 3 UPS in AD

### 3.1 Aβ and UPS

**Figure 3:**
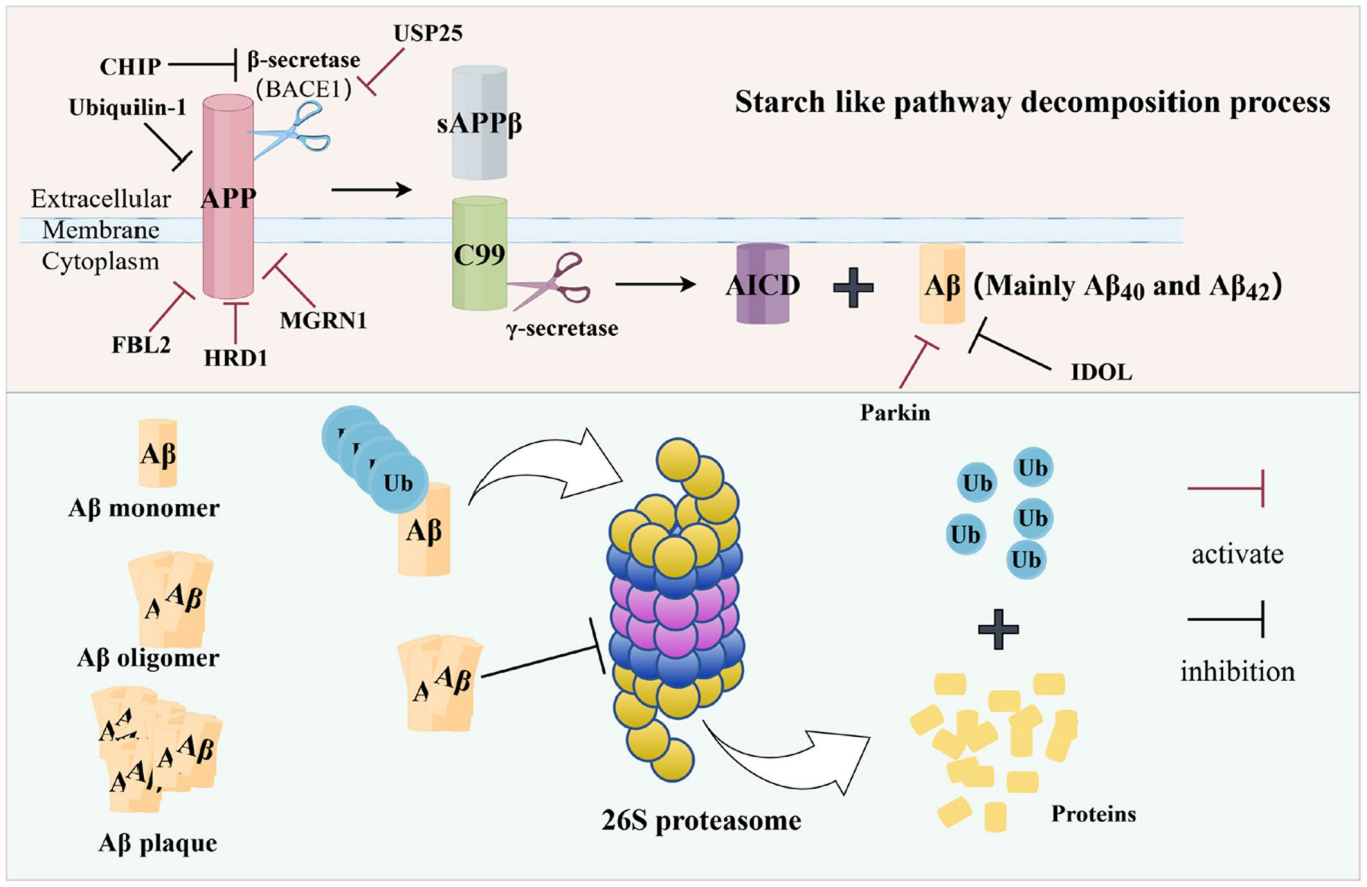
Aβ and UPS (In AD, APP is cleaved by β-secretase and *γ*-secretase to form Aβ, especially Aβ_40_ and Aβ_42_, respectively, leading to the accumulation of toxic proteins. UPS can degrade excess proteins, but when Aβ is produced in large quantities to form oligomers, Aβ in turn affects the proteasome activity, which leads to the malfunctioning of the UPS, as shown in the figure. A number of UPS-related enzymes participate in these processes, promoting the breakdown or aggregation of APP and Aβ. The figure was drawn using Figdraw).

In AD, Aβ and UPS interact with each other in a causal manner (Figure 3). On the one hand, UPS has the effect of inhibiting Aβ production and promoting Aβ degradation; on the other hand, Aβ can, in turn, react with UPS and impede the function of UPS by inhibiting proteasome activity (Oh et al., 2005).

The UPS suppresses Aβ pathogenesis through two primary mechanisms: targeting its precursor (APP/BACE1) for degradation and directly clearing Aβ peptides. Several E3 ubiquitin ligases are central to this process. For instance, CHIP promotes the ubiquitination and degradation of BACE1 (β-secretase), thereby reducing APP processing and Aβ generation (Singh and Pati, 2015). Similarly, HRD1, FBL2, and Parkin facilitate the ubiquitination and proteasomal degradation of APP or its C-terminal fragments (CTFs), limiting the substrate available for Aβ production (Kaneko et al., 2010). The LDL receptor-inducible degradator (IDOL) represents another E3 ligase whose ablation has been shown to promote Aβ clearance and improve cognitive function in APP/PS1 mice (Choi et al., 2015). Conversely, the deubiquitinating enzyme USP25 counteracts this process by stabilizing BACE1, enhancing APP cleavage, and increasing Aβ production (Zheng et al., 2022). The proteasome also directly degrades Aβ peptides, as evidenced by the finding that proteasome inhibition significantly reduces Aβ_42_ clearance (Lopez Salon et al., 2003).

However, this protective UPS function is compromised by Aβ itself, which instigates a vicious cycle by directly impairing the proteasome. Aβ oligomers inhibit the hydrolytic activity of the 26S proteasome, leading to widespread UPS dysfunction (Almeida et al., 2006). Furthermore, Aβ_42_ promotes the expression of E1 and the accumulation of ubiquitinated proteins, creating a proteostatic burden that further overwhelms the already impaired degradation system (Hong et al., 2014). The loss of other protective UPS components, such as Ubiquilin-1 and MGRN1, in the AD brain exacerbates this cycle, leading to increased APP stability and Aβ deposition (Natunen et al., 2016).

### 3.2 Tau and UPS

**Figure 4:**
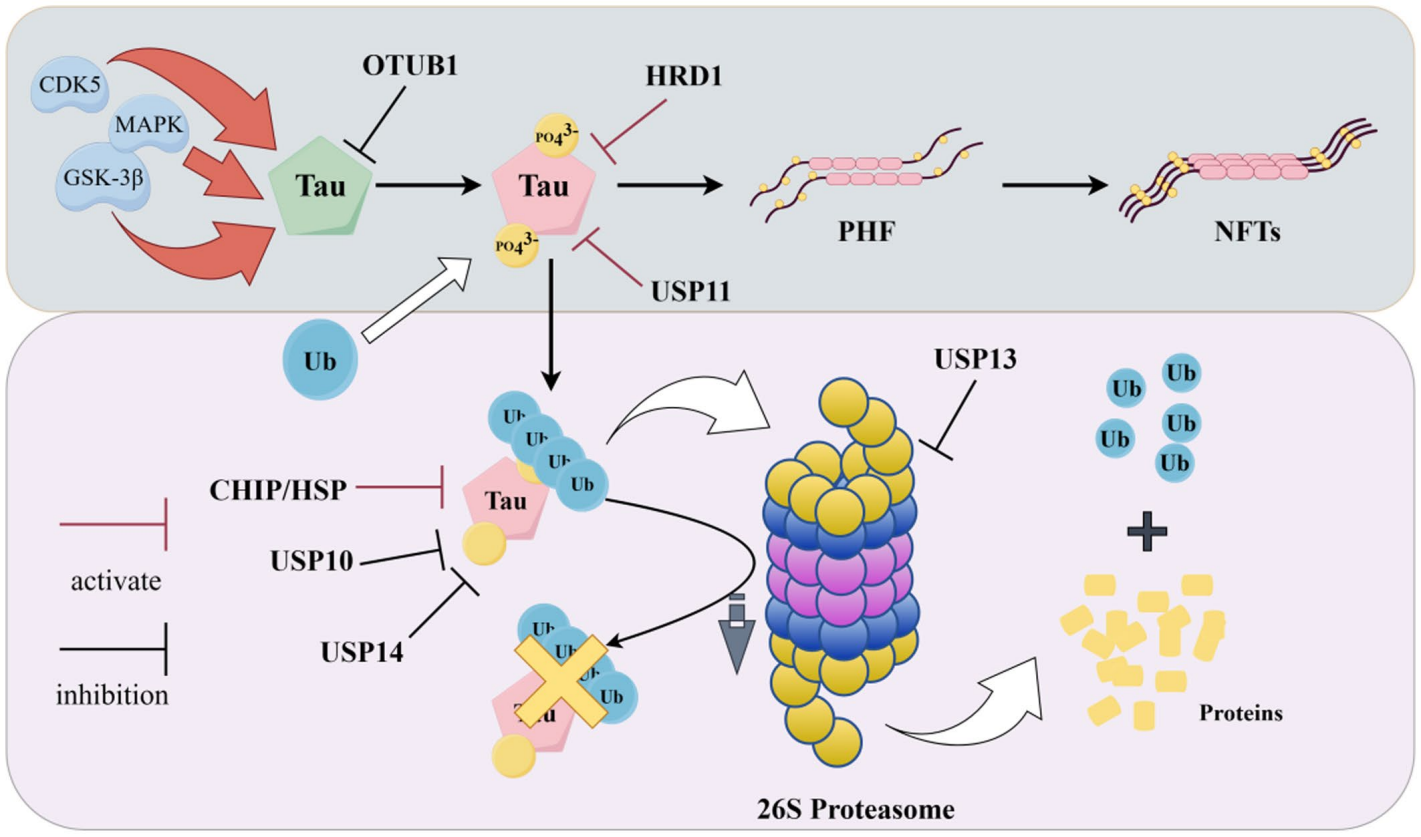
Tau and UPS (In pathological conditions, Tau proteins are abnormally phosphorylated and form neurofibrillary tangles after excessive accumulation. Ubiquitin ligases and deubiquitinating enzymes degrade misfolded proteins. Ubiquitin ligases and deubiquitinating enzymes promote or inhibit this process, as shown in the figure. In addition, ubiquitinated Tau proteins cannot be degraded when proteasome activity is decreased. The figure was drawn using Figdraw).

Over-phosphorylated Tau proteins accumulate in neurons, which can cause a series of typical pathological features of AD, such as abnormal synaptic function, neuronal deficits, and mitochondrial disruption (Van der Jeugd et al., 2012). Previous findings have shown that abnormal UPS function is closely related to the phosphorylation and accumulation of Tau protein (Ciechanover and Kwon, 2015) (Figure 4).

The stability of Tau protein is critically regulated by E3 ubiquitin ligases, whose dysfunction contributes directly to its pathological accumulation in AD. For instance, the CHIP/HSP70 complex is essential for ubiquitinating misfolded Tau, and its deficiency in AD models leads to the accumulation of hyperphosphorylated Tau (Nadel et al., 2023). Similarly, the E3 ligase HRD1 serves a protective role, as its expression is negatively correlated with Tau pathology in the hippocampi of AD patients, and its knockdown promotes the accumulation of hyperphosphorylated Tau (Potjewyd and Axtman, 2021).

Beyond E3 ligases, DUBs play an equally critical role in AD by antagonizing the ubiquitination of Tau, thereby stabilizing it and promoting its pathological accumulation. A cohort of ubiquitin-specific proteases (USPs) has been mechanistically linked to tauopathy. For instance, USP10 overexpression elevates both total and phosphorylated Tau levels, inducing aggregation and delaying its degradation (Wei et al., 2022). Similarly, USP11 promotes the deubiquitination of Tau at Lys281, enhancing its hyperphosphorylation and aggregation—a process that may contribute to the observed sex differences in Tau pathology, given the higher levels found in females (Yan et al., 2022). Conversely, genetic knockdown of USP13 has been shown to enhance proteasome activity, increase ubiquitination of phosphorylated Tau, and facilitate its clearance (Liu et al., 2019). The proteasome-associated USP14 also operates as a negative regulator of Tau degradation, as its inhibition accelerates the clearance of hyperphosphorylated Tau (Wang D. et al., 2021). Other DUB families contribute to this regulatory network. OTUB1 directly removes K48-linked polyubiquitin chains from Tau, preventing its proteasomal degradation and accelerating its hyperphosphorylation and oligomerization in TauP301S mouse models (Liu et al., 2022). Ubiquitin carboxy-terminal hydrolase L1 (UCH-L1), also known as Parkinson’s disease protein 5, has the functions of processing ubiquitin precursors, hydrolyzing ubiquitinated protein residues, and maintaining the level of free ubiquitin monomer in the brain. In Parkinson’s disease, mutated UCHL1 causes a variety of pathological conditions: protein degradation caused by loss of catalytic function is blocked, abnormal protein interaction affects mitochondrial autophagy, molecular chaperone-mediated autophagy, etc., and oxidatively modified UCHL1 aggregates to form a pathological protein network (Buneeva and Medvedev, 2024). In addition, the expression of UCH-L1 is inversely correlated with NFT density in AD brains, and its inhibition promotes NFT formation, underscoring its role in Tau homeostasis (Graham and Liu, 2017).

It has been found that in the brains of AD patients, proteasome activity is reduced, and this change is negatively correlated with the number of NFTs, suggesting that inhibition of proteasome activity prevents the degradation of phosphorylated Tau proteins, which in turn promotes the formation of NFTs (Harris et al., 2020). Della et al. (2002) found that the addition of purified non-ubiquitylated recombinant Tau to the 20S proteasome resulted in the degradation of Tau proteins, indicating a UPS-dependent degradation of Tau. Inhibition of proteasome function in 3xTgAD mice was found to lead to pathological accumulation of Tau (Tseng et al., 2008). In addition, changes in the co-localization of excess p-Tau with UbK48 were found in hippocampal neurons of AD model mice, and both led to cellular inflammation and induced apoptosis in neuronal cells (Li et al., 2024).

### 3.3 Synapse and UPS

**Figure 5:**
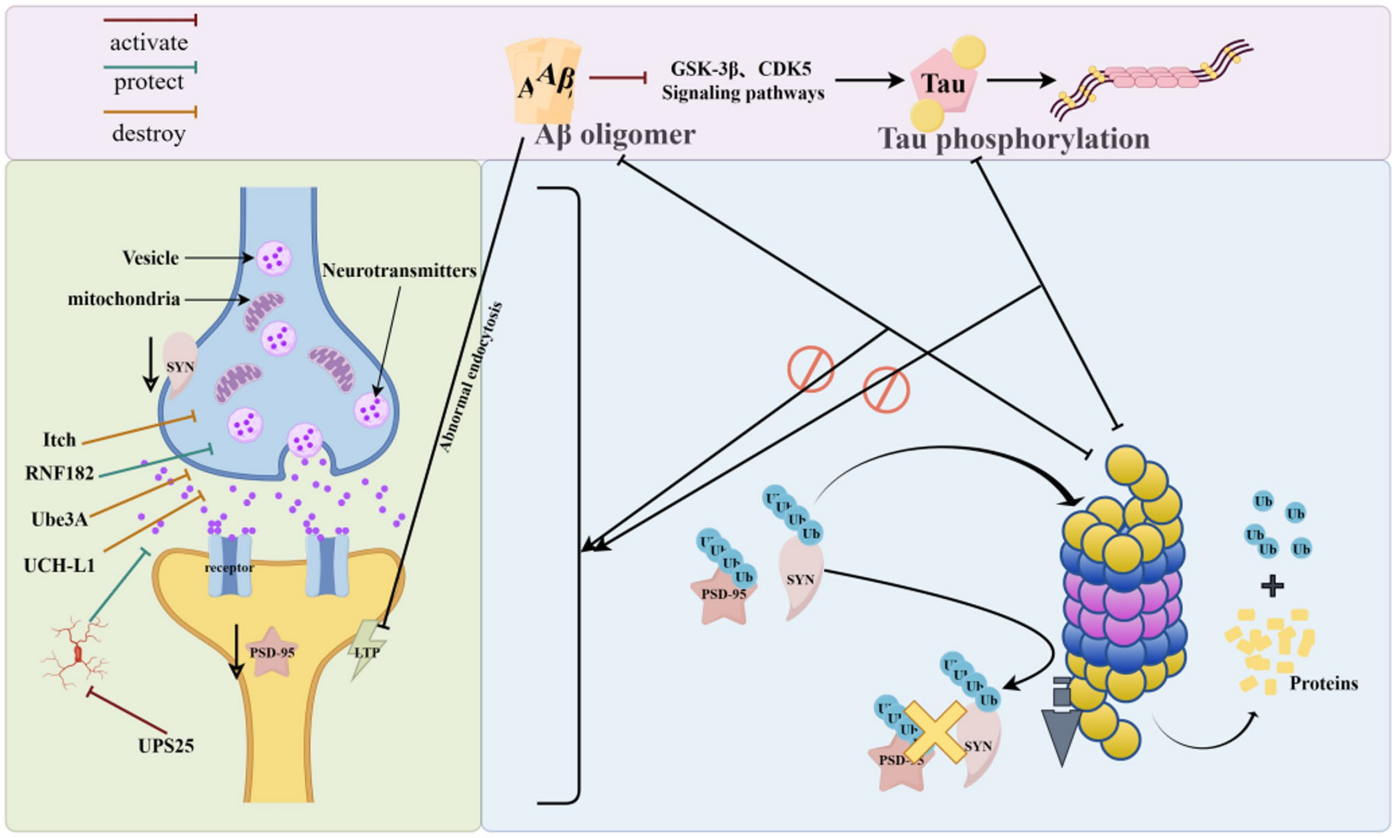
Synapse and UPS (UPS can regulate synaptic protein turnover by ubiquitination of mislabeled proteins, removing abnormally aggregated Aβ and phosphorylated Tau from synapses, and conversely, abnormally aggregated Aβ and phosphorylated Tau can disrupt proteasomal activity and interfere with the process of ubiquitination, leading to synaptic dysfunction. As shown in the figure, UPS-related enzymes can regulate the accumulation and degradation of synaptic proteins, which in turn affect synaptic function. The figure was drawn using Figdraw).

UPS plays a central role in the physiological activity of synapses. UPS exerts a role in protein degradation, which in turn regulates the synaptic plasticity in neurons and maintains neuronal activity, and signaling between neurons is dependent on the regulation of UPS (Kumari et al., 2017) (Figure 5).

Abnormal UPS function can lead to changes in synaptic structure, function, and synapse-associated proteins, affecting neuronal homeostasis and signaling function, which can cause neuronal damage and reduced learning and memory ability (Audrain et al., 2016). Post-synaptic dense protein 95 (PSD-95) expression levels are reduced in AD patients and AD model mice (Savioz et al., 2014; Hondius et al., 2016). UPS can regulate PSD-95 metabolism and is important for regulating the protein environment in the postsynaptic dense zone of neurons (Caldeira et al., 2014).

It was found that ATP6V0C is a substrate of RNF182; the up-regulation of RNF182 in AD brain can be mediated by the proteasomal degradation pathway to promote ATP6V0C degradation, which ultimately leads to neuronal apoptosis and accelerates AD pathology (Okamoto et al., 2020). TAp73 regulates the expression of microRNA-34a. microRNA-34a can promote neuronal differentiation and neurite growth. In AD, Aβ_42_ can induce Itch hyperphosphorylation by pathologically activating the c-Jun amino-terminal kinase signaling pathway, and the aberrantly modified Itch will further degrade TAp73 in a ubiquitinated form, which alters the expression of neuronal cell-cycle proteins, ultimately leading to neuronal death and accelerating the progression of AD (Chauhan et al., 2020). It was found that Ube3A expression in the Tg2576 mouse brain declined with age, and the decrease in Ube3A levels caused the accumulation of synaptotoxic proteins, which in turn induced a decrease in glutamate receptors on the synaptic surface, ultimately leading to abnormal synaptic function (Olabarria et al., 2019).

Zheng et al. (2021) found that overexpression of USP25 promotes microglia activation while inducing synaptic and cognitive deficits, whereas inhibition of the USP25 gene reduces neuroinflammation and improves synaptic and cognitive function in 5 × FAD mice. It was found that UCH-L1 levels were decreased in the brain of AD model animals, and up-regulation of UCH-L1 levels repaired synaptic function and memory loss in this mouse (Tramutola et al., 2016).

### 3.4 AD risk factors and UPS

Although aging is the main cause of UPS dysfunction, more and more evidence shows that UPS may be the key convergence point of a variety of risk factors leading to AD. Clarifying the relationship between these risk factors and UPS is important for identifying high-risk groups and developing preventive strategies.

#### 3.4.1 Lack of sleep

Circadian rhythms set a schedule for brain protein clearance. Sleep is an important “window period” for the brain to clear abnormal proteins. UPS plays an important role in this process. An experiment in Drosophila showed that the periodic accumulation and degradation of core clock protein depended on UPS, and sleep deprivation could inhibit the activity of proteasome, lead to the dysfunction of E3 ubiquitin ligase, and then aggravate neuronal damage (Musiek and Holtzman, 2016). In AD, sleep deprivation can aggravate the accumulation of Aβ and tau proteins in the brain, leading to UPS dysfunction (Ukita et al., 2022). Therefore, it can be considered that sleep deprivation is a continuous state of UPS function inhibition and is an important intervention risk factor for AD.

#### 3.4.2 Traumatic brain injury

Post traumatic brain injury (TBI) is a kind of strong physical and chemical shock, which will cause acute and severe cell folding and stress reaction in neurons, promote a large number of protein misfolding, and bring huge “cleaning” pressure to UPS system (Wang K. K. et al., 2017). In addition, TBI can induce oxidative stress and other reactions, hinder the degradation process of the proteasome, and inhibit the enzyme activity of UCH-L1. In the TBI animal model, extensive protein aggregation and ubiquitinated protein accumulation were found, which further explained the dysfunction of UPS caused by TBI (Mi et al., 2021). To sum up, TBI can inhibit the activity of UCH-L1 and other functional proteins, destroy the normal function of UPS, and then lead to the clearance barriers and abnormal aggregation of key pathogenic proteins of AD, and ultimately increase the risk of AD.

#### 3.4.3 Cerebrovascular factors

Cerebrovascular factors such as hypertension and stroke are important risk factors for AD. Cerebrovascular diseases destroy the energy supply of the brain, keeping the tissue in a low perfusion state for a long time, and even directly cause a series of reactions, such as oxidative stress and neuritis. This further affected the function of UPS and reduced its ability to clear abnormal proteins in the brain. On the one hand, the transcription factor HIF-1α cannot be degraded under hypoxic conditions. High levels of HIF-1α will enter the nucleus and strongly upregulate the gene expression of BACE1, leading to more Aβ production and aggregation (Lonati et al., 2014). On the other hand, the overall functional efficiency of UPS decreased, and the abnormally phosphorylated tau protein could not be removed in time, and finally aggregated to form NFTs.

#### 3.4.4 ApoE4 genotype

ApoE4 is the strongest genetic risk factor for AD. It plays a central role in the pathogenesis of AD by aggravating the imbalance of protein homeostasis. The main mechanism is that apoE4 not only directly increases the clearance burden of UPS by reducing Aβ clearance, promoting its fibrosis aggregation, and inducing tau protein hyperphosphorylation to produce a large number of abnormal proteins; At the same time, these accumulated neurotoxic proteins (such as Aβ oligomers) will reverse inhibit the function of UPS, forming a self-reinforcing vicious circle. In addition, oxidative stress is a key early event in the brain of apoE4 carriers. Inhibiting the activity of UCH-L1 and other deubiquitinates leads to a decrease in ubiquitin recovery efficiency and further weakens the degradation ability of UPS. The overall decline of the UPS function eventually leads to the elimination of toxic proteins, thus accelerating the process of neurodegenerative disease (Mi et al., 2021).

## 4 Current status of AD therapy targeting UPS components

Pathological changes in UPS are associated with a variety of diseases, including neurodegenerative diseases, malignant tumors, hereditary diseases, inflammation, cardiovascular diseases, etc. (Pohl and Dikic, 2019). In recent years, some progress has been made in the development of drugs targeting UPS, and some drugs have entered the clinical research stage (Zhang et al., 2020). Among them, AD has been receiving attention from a large number of researchers. A large number of studies have provided brand new ideas and prospects for the treatment of AD.

### 4.1 Modulation of the proteasome for the treatment of AD

Proteasomal dysfunction is a hallmark of AD, making its functional restoration a compelling therapeutic strategy. Current approaches can be categorized based on their primary mode of action: direct activation of the proteasome, indirect restoration of proteostasis, and the paradoxical use of inhibitors for target stabilization (Table 1).

**Table 1:** Experimental study of targeting the proteasome in the treatment of AD.

Therapeutic drugs or methods	Experimental subjects	Model species	Key indicators of the AD model	Affected pathways/genes/proteins	References
Ursolic acid	*Caenorhabditis elegans*	*Caenorhabditis elegans*	Aβ↓, Aβ oligomer↓	-	Wang et al. (2022)
Cilostazol	Mice	Double-transgenic rTg4510 mice	Tau↓	cAMP/PKA pathway	Schaler and Myeku (2018)
Resveratrol	Mice	Transgenic 3xTg-AD mice	Aβ↓, Tau↓	SIRT1 pathway	Corpas et al. (2018)
		Transgenic 5XFAD mice	Aβ↓, Tau↓	-	Sarroca et al. (2021)
SLAB1	Mice	Transgenic 3xTg-AD mice	Aβ↓, Pro-inflammatory factors↓	Gut microbiota	Bonfili et al. (2017)
Ghrelin	Cells	SH-SY5Y	Aβ↓	-	Cecarini et al. (2015)
BTZ	Mice	Tg2576 transgenic mice	Aβ↓	P-gp protein	Vulin et al. (2023)
Lactacystin	Mice	APP/PS1 transgenic mice	LTP↑, Synaptic-associated proteins↑	mTOR pathway	Krishna et al. (2020)
18α-GA **+** n-3FA	Mice	Transgenic 5XFAD mice	Aβ↓	Nrf2 factor	Mladenovic Djordjevic et al. (2021)

“↑” denotes increased levels; “↓” denotes reduced levels; “-” indicates that the article did not mention.

#### 4.1.1 Direct proteasome activators

This class of compounds directly enhances the catalytic activity or expression of the proteasome, facilitating the clearance of misfolded proteins.

Ursolic acid is a natural triterpenoid with pharmacological effects such as antioxidant, anti-inflammatory, and neuroprotective. Using ursolic acid to intervene in an Aβ-induced transgenic AD pathology model of Cryptobacterium hidradii nematode, it was found that ursolic acid prevented amyloid β-induced proteotoxic stress by enhancing *in vivo* proteasome activity, transcriptionally augmenting the UPS, and thereby reducing the levels of amyloid β monomers, oligomers, and deposits. In contrast, the proteasome inhibitor MG132 eliminated the therapeutic effect of ursolic acid (Wang et al., 2022).

Cilostazol is an FDA-approved phosphodiesterase 3 (PDE3) inhibitor, which is mainly used to treat intermittent claudication symptoms, and possesses pharmacological effects such as vasodilatation, protection of vascular endothelial cells, and inhibition of platelet aggregation. In addition, Cilostazol has been investigated for the improvement of clinical symptoms in patients with mild cognitive impairment, as it shows potential to improve cognitive function (Tai et al., 2017). Researchers explored the effects of Cilostazol on a mouse model of Tau proteinopathy and found that Cilostazol significantly improved spatial learning and memory abilities in mice, that Cilostazol significantly increased the activity of the 26S proteasome, which reduced the accumulation of abnormal Tau proteins, and that this effect was closely linked to the cAMP/PKA pathway (Schaler and Myeku, 2018).

#### 4.1.2 Indirect restoration of proteasome homeostasis

These interventions counter upstream insults that cause proteasomal impairment, such as oxidative stress and neuroinflammation, thereby indirectly restoring its function.

Resveratrol is a polyphenol that has been shown to have antioxidant, anti-inflammatory, neuroprotective, and anti-aging pharmacological effects (Novelle et al., 2015). It was found that resveratrol could improve the learning and memory ability of AD model animals by activating proteasome activity, and then promote the clearance of Aβ and Tau protein in the brain of AD transgenic animals, thus exerting neuroprotective effects (Corpas et al., 2018). In a study by Sarroca et al. (2021) it was found that a high-fat diet caused AD-like neuropathological manifestations in wild-type mice and exacerbated the condition in 5XFAD mice. At the molecular level, the high-fat diet increased chymotrypsin-like and cysteine proteasomes in the cerebral cortex of WT and 5XFAD mice, like proteasome and 20S proteasome catalytic β5 subunit levels in the cerebral cortex of WT and 5XFAD mice, and led to increased amyloid and Tau protein deposition. And resveratrol can reverse the above situation, restore the abnormal proteasome homeostasis, reduce the deposition of toxic proteins in the animal’s brain, and restore the impaired memory ability and reduce abnormal behavioral manifestations such as anxiety and fear.

Recent studies have shown that intestinal flora can affect the cognitive function of AD patients through the “microbe-gut-brain” axis, and an imbalance of intestinal flora may lead to peripheral inflammation, inflammatory factors diffuse into the brain through the blood–brain barrier, which activates the neuroimmune response in the brain, and the excessive release of inflammatory factors causes neurotoxicity, which in turn leads to cognitive impairment (Chevalier et al., 2025). SLAB51 is a formulation of nine live strains of bacteria, including Lactobacillus and Bifidobacterium, which are commonly considered probiotics. It was found that SLAB51 improved the structure of intestinal flora and regulated the concentration of pro-inflammatory cytokines in plasma in AD model mice, while at the same time, SLAB51 restored the impaired proteasome activity in AD model mice, which in turn facilitated the degradation of toxic proteins in the brain and improved the cognitive function of the mice (Bonfili et al., 2017).

Growth hormone-releasing peptide (Ghrelin), a new endogenous brain-gut peptide consisting of 28 amino acids, has a role in inhibiting insulin secretion, regulating blood glucose values, and has neuroprotective and anti-apoptotic activity in neurological diseases. In a study, Ghrelin was found to have a regulatory effect on UPS. The study used SH-SY5Y neuroblastoma cells, which were treated with Ghrelin, and found that Ghrelin had cell proliferative and anti-apoptotic effects that promoted cell survival, and Ghrelin also promoted the activation of the proteasome system, which, in turn, facilitated the clearance of toxic protein aggregates from the cells. In addition, Ghrelin affected the interaction between UPS and autophagy, providing further support for neuronal cell homeostasis (Cecarini et al., 2015).

#### 4.1.3 Proteasome inhibitors in AD: a paradoxical approach

Interestingly, proteasome inhibitors can be leveraged to stabilize specific neuroprotective proteins by preventing their degradation, offering an alternative strategy.

P-glycoprotein (P-gp) is highly expressed in endothelial cells of the blood–brain barrier. Its core function is to pump substrates out of the cells into the blood. It is a key transporter for clearing Aβ in the brain. In AD, excessive aggregation of Aβ will activate ubiquitin ligase, promote the ubiquitination of P-gp, and degrade it through proteasome, which will lead to the decline of P-gp expression and function on the blood–brain barrier, and further weaken the ability of Aβ clearance, forming a vicious circle (Chai et al., 2020). However, deletion of P-gp at the blood–brain barrier was found in the brains of AD model animals. In addition, Aβ_40_ can trigger P-gp ubiquitination and degradation via the UPS (Hartz et al., 2016). Vulin et al. (2023) found that after treatment with the proteasome inhibitor bortezomib (BTZ) targeting the proteasome, BTZ inhibited proteasomal activity and could benefit the treatment of AD by blocking the degradation of P-gp, which could in turn inhibit the level of Aβ expression in the brain.

In AD, impaired UPS function leads to the abnormal accumulation of Aβ, Tau, and other proteins, which in turn affects the structure and function of synapses, leading to synaptic damage, which in turn affects the ability of learning and remember. MG132 and lactacystin are two proteasome inhibitors, and the study of Krishna et al. (2020) found that MG132 and lactacystin can upregulate the mTOR pathway by upregulating the related protein synthesis, which in turn promotes the synthesis of synapse-related proteins, and can restore impaired LTP and synaptic labeling and trapping phenomena in the hippocampus of AD model animals.

#### 4.1.4 Multi-target and combination strategies

Combination therapies can simultaneously engage multiple pathways to activate the UPS.

Combined drug administration can promote the activation of the ubiquitin-proteasome through synergistic effects, thereby improving the pathological phenotypes related to AD. Mladenovic Djordjevic et al. (2021) used a 5xFAD transgenic mouse model with 18 alpha-glycyrrhetinic acid (18α-GA) co-administered with omega-3 fatty acids (n-3FA) co-administration. The results of the study showed that the co-administration significantly improved motor function, cognition, anxiety level, and frailty in the AD model animals, as well as reduced Aβ plaque load in the brain.

### 4.2 Modulation of ubiquitin ligase for the treatment of AD

E3 ubiquitin ligases confer specificity to the ubiquitination process, making them attractive yet challenging therapeutic targets. Strategies to modulate their activity, either by enhancing the function of protective ligases or inhibiting detrimental ones, are highlighted below (Table 2).

**Table 2:** Experimental study of targeting ubiquitin ligase in the treatment of AD.

Therapeutic drugs or methods	Experimental subjects	Model species	Key indicators of the AD model	Affected pathways/genes/proteins	References
SFN	Mice	Transgenic 3xTg-AD mice	Aβ↓, Tau↓	-	Lee et al. (2018)
XBP-1x	Cells	HEK293, SH-SY5Y	BACE1↓, Aβ↓	-	Gerakis et al. (2016)
Geniposide	Cells	Primary cortical neurons	APP↓	IRE1α	Cui et al. (2018)
ASO	Mice	APP/PS1 transgenic mice	Aβ↓	Microglia	Gao et al. (2023)

“↓” denotes reduced levels; “-” indicates that the article did not mention.

#### 4.2.1 Upregulation of cytoprotective E3 ligases

Enhance the expression or activity of E3 ligase and promote the degradation of AD-related pathological proteins.

Radish thiols (SFN) are mainly extracted from cruciferous plants, including cauliflower and broccoli, which are a class of purely natural compounds with good anti-inflammatory and antioxidant functions, and are clinically used in the treatment of cancer, diabetes, and other diseases (Zhang et al., 2022). Lee et al. (2018) showed that SFN could reduce the accumulation of neurotoxic proteins in the brains of triple transgenic AD mouse models and improve the learning and memory abilities of mice by up-regulating CHIP protein levels.

Deficiency of XBP-1 s leads to increased Aβ-related toxicity, whereas expression of XBP-1 s restores synaptic plasticity and memory control. HRD1 is involved in endoplasmic reticulum-associated degradation pathways and is responsible for labeling misfolded Aβ_42_ oligomers upregulate HRD1 expression through an XBP-1 s-dependent pathway, and HRD1, as a ubiquitin ligase, decreases BACE1 expression and activity, thereby reducing Aβ_42_ oligomer production and associated toxicity, and thus alleviating the associated pathology. Studies have shown that XBP-1 s indirectly reduce the expression and activity of BACE1 by upregulating HRD1 expression. In addition, XBP-1 s indirectly affect BACE1 by regulating HRD1 expression at the transcriptional level (Gerakis et al., 2016). In addition, geniposide can also promote APP clearance by up-regulating HRD1 expression, which in turn promotes APP clearance; specifically, geniposide induced and amplified HRD1 expression in cortical neurons cultured with high glucose, which reduced APP loading in neurons, re-established protein homeostasis, and restored damaged neuronal function (Cui et al., 2018).

#### 4.2.2 Inhibition of pathogenic E3 ligases

An alternative approach is to inhibit E3 ligases that negatively regulate beneficial pathways, such as those involved in Aβ clearance.

Low-density lipoprotein receptor (LDLR) and low-density lipoprotein receptor-related protein 1 (LRP1) are major regulators of ApoE metabolism and mediate the uptake and degradation of ApoE-containing lipoprotein particles by brain cells. Idol is a negative regulator of LDLR in microglia, and the absence of Idol in microglia increases the level of LDLR protein, which in turn promotes the uptake and clearance of ApoE and Aβ by microglia (Choi et al., 2015). Gao et al. (2023) therapeutically inhibited IDOL activity in the brains of AD model mice using antisense oligonucleotides (ASO) and found that the inhibition of IDOL activity resulted in a significant reduction of Aβ deposition in the brains of AD mice, upregulation of lysosomal/phagocytosis genes in microglia, and improved cognitive function of AD mice by ASO by improving the ability of improved spatial learning and memory in AD model mice.

### 4.3 Modulation of deubiquitinating enzymes for the treatment of AD

DUBs counteract the ubiquitination process, and their dysregulation contributes to AD pathogenesis. Inhibiting specific DUBs to promote the degradation of pathological proteins has emerged as a viable therapeutic strategy (Table 3).

**Table 3:** Experimental study of targeted deubiquitinase in the treatment of AD.

Therapeutic drugs or methods	Experimental subjects	Model species	Key indicators of the AD model	Affected pathways/genes/proteins	References
AZ1	Mice	transgenic 5XFAD mice	Aβ↓, synaptic↑	Microglia	Zheng et al. (2021), Zheng et al. (2022)
USP14-1, USP14-2, USP14-3	Cells	HEK293-trex-htau40	Tau↓	oxidative stress	Lee et al. (2015)
IU1-47	Mice	Transgenic 5XFAD mice, transgenic 3xTg-AD mice	Tau↓	-	Boselli et al. (2017)
overexpression of UCH-L1	Mice	APP23/PS45 transgenic mice	Aβ↓	-	Zhang et al. (2014)

“↑” denotes increased levels; “↓” denotes reduced levels; “-” indicates that the article did not mention.

#### 4.3.1 Inhibition of USP25 to ameliorate Aβ pathology and neuroinflammation

USP25 exacerbates AD through dual pathways, making it a high-value target.

Overexpression of USP25 encoded by chromosome 21 significantly increased amyloid deposition in 5 × FAD mice, and USP25 deficiency ameliorated amyloid pathology by regulating APP processing and Aβ production. Inhibition of USP25 expression using compound AZ1 significantly reduced amyloid plaque burden in 5 × FAD mice and improved cognitive performance in AD model mice (Zheng et al., 2022). In addition, Zheng et al. (2021) found that overexpression of USP25 leads to microglia activation and inflammation, further induces synaptic and cognitive deficits, and silencing of the USP25 gene attenuates the neuroinflammatory response induced by microglia activation, restores microglia homeostasis, and improves synaptic elimination, which then restores cognitive function in AD model animals.

#### 4.3.2 Inhibition of USP14 to enhance proteasomal clearance

Targeting the proteasome-associated DUB USP14 is a strategy to boost overall proteasome activity and facilitate the clearance of aggregation-prone proteins.

SELEX technology is an experimental method for enriching high-affinity and high-specificity nucleic acid aptamers from a large number of random sequences after multiple rounds of screening. Aptamers, also known as “chemical antibodies,” are biomolecules that can bind to a number of different targets and are characterized by their small molecular weight, ease of synthesis and modification, and adjustable stability. Lee et al. (2015) screened three RNA aptamers, called USP14-1, USP14-2, and USP14-3, which can specifically bind USP14 by SELEX technology, and showed that these RNA aptamers can effectively inhibit the deubiquitylation activity of USP14 and enhance the proteasome activity. IU1-47 is a potent and selective inhibitor of USP14, and inhibition of the deubiquitinating activity of USP14 can facilitate the degradation of the speciated substrate by enhancing proteasome activity and thereby promoting degradation. In a study by Boselli et al. (2017) IU1-47 was found to show the ability to reduce endogenous wild-type Tau protein levels in cell culture to further maintain intracellular protein homeostasis and restore damaged neuronal function.

#### 4.3.3 Overexpression of UCH-L1 to promote BACE1 degradation

In contrast to inhibition, enhancing the activity of certain DUBs can also be beneficial, as exemplified by UCH-L1.

Zhang et al. (2014) used APP23/PS45 double transgenic AD model mice, overexpressing UCH-L1 by intracerebral injection of UCH-L1 expressing recombinant adeno-associated virus, and the results showed that UCH-L1 overexpression could regulate the degradation of BACE1, which in turn reduced the production of APP CTF and the level of Aβ, inhibited the formation of neuritic plaques, and ameliorated the memory deficits in the AD transgenic model mice to slow down the progression of AD.

### 4.4 Holistic regulation of the UPS system for the treatment of AD

Beyond targeting specific UPS components, a complementary strategy involves holistic regulation through upstream signaling pathways and cellular processes. These interventions restore UPS function indirectly by activating cytoprotective programs, enhancing protein folding capacity, or modulating UPS-related gene expression (Table 4).

**Table 4:** Experimental study on the treatment of AD by regulating UPS.

Therapeutic drugs or methods	Experimental subjects	Model species	Key indicators of the AD model	Affected pathways/genes/proteins	References
24-OHC	Cells	SK-N-BE neuroblastoma cells	Tau↓	SIRT1/PGC1α/Nrf2 pathway	Testa et al. (2023)
Cbx	Rat	SD rats	APP↓, BACE1↓, Tau↓	-	Sharma et al. (2020)
SELENOW	Mice	Transgenic 3xTg-AD mice	Tau↓	Hsp70	Zhang and Song (2021)
YZS	Rat	SD rats、	p-Tau↓	-	Li et al. (2021)
IOP	Mice	Transgenic 3xTg-AD mice	Aβ↓	-	Wang et al. (2023)

“↓” denotes reduced levels; “-” indicates that the article did not mention.

#### 4.4.1 Activation of cytoprotective and antioxidant pathways

This approach engages master regulatory pathways that transcriptionally upregulate a network of genes, including those within the UPS, to combat proteostatic stress.

24-Hydroxycholesterol (24-OHC) has been found to be neuroprotective, with significantly reduced levels of 24-OHC in the frontal and occipital cortices in the AD brain. The results of *in vitro* experiments showed that up-regulation of 24-OHC levels in an in vitro model of AD was beneficial in preventing the accumulation of over-phosphorylated Tau proteins in cells and slowing down the progression of AD (Testa et al., 2018). A study by Testa et al. (2023) further found that in SK-N-BE neuroblastoma cells, 24-OHC could inhibit the formation of NFT and reverse neuronal death through its action on the SIRT1/PGC1α/ Nrf2 signaling pathway, promote the activation of the UPS system, and inhibit the excessive accumulation of Tau protein through the ubiquitination and degradation function of UPS, which in turn prevents the formation of NFTs, reverses neuronal death, and exerts neuroprotective effects.

#### 4.4.2 Enhancement of molecular chaperone systems

Boosting the cellular chaperone network aids in the recognition and presentation of misfolded proteins to the UPS, thereby improving the efficiency of substrate degradation.

Carbenoxolone (Cbx), a heat shock protein inducer derived from licorice, is commonly used clinically in the treatment of gastric ulcers (Hellmich et al., 2013). It was found that Cbx was able to upregulate mRNA and protein expression levels of molecular chaperones and restore the expression of HSPs that declined after Aβ_42_ oligomer injection. Upregulation of proteasome activity promoted the restoration of UPS function. Aβ_42_ oligomer injection resulted in a significant increase in ubiquitin mRNA and protein expression levels, and Cbx co-treatment significantly reduced these increases. Meanwhile, Cbx co-treatment significantly suppressed the mRNA and protein expression levels of APP, Tau, and BACE1 in the brains of AD model animals, suggesting that Cbx is important for combating the pathological progression of AD (Sharma et al., 2020).

Selenoprotein W (SELENOW), a small protein sensitive to changes in selenium with antioxidant properties, is closely associated with Tau protein accumulation in AD, and can reduce Tau protein accumulation by promoting Tau protein ubiquitination. SELENOW deficiency leads to dysregulation of Tau proteins, synaptic deficits, and impaired LTP, which in turn causes memory deficits (Zhang and Song, 2021). Ren et al. (2024) showed that overexpressed SELENOW competes with HSP70 for Tau interactions and promotes its degradation via UPS, which in turn ameliorates memory impairment and Tau protein-related pathology and delays the progression of AD in AD model mice.

#### 4.4.3 Multi-component natural formulations and extracts

Certain natural products exert their effects through a combination of mechanisms, leading to a broad upregulation of the UPS cascade.

Yuan Zhi San is a classical Chinese medicine compound formula, which has the effect of benefiting the intellect, resolving phlegm and opening up the mind. The study of Li et al. (2021) found that, Yuan Zhi San could up-regulate the expression levels of ubiquitin-related molecules and the 26S proteasome in the brains of the AD model animals, and restored the impaired UPS enzyme cascade reaction, which in turn, through the restoration of the UPS activity, promoted the degradation of hyperphosphorylated Tau protein in the brain of AD rats, which in turn improved the learning and memory ability of AD model animals and inhibited the further development of AD.

Inonotus obliquus polysaccharide (IOP) is one of the main components of Inonotus obliquus, and it is commonly used clinically in diabetes mellitus therapy (Ding et al., 2024). Diabetes mellitus is widely recognized as one of the important factors contributing to the development of AD, and some scholars refer to AD as type III diabetes mellitus (Colin et al., 2023). Wang et al. (2023) found that IOP could significantly increase the expression levels of ubiquitin, E1, Parkin, and other ubiquitin-associated enzymes in the hippocampus of mice in a transgenic model of AD, which, in turn, increased the activity of the UPS, reduced the accumulation of amyloid aggregates, and reduced the number of AD-related symptoms.

### 4.5 Targeted protein degradation technology for the treatment of AD

Protein hydrolysis-targeted chimeras (PROTACs) are a UPS-based targeted protein degradation technology, the core of which is designed as a bifunctional small-molecule chimera consisting of a target protein ligand (which specifically binds to the target protein, POI), E3 ubiquitin ligase ligand (which recruits a specific E3 ubiquitin ligase), and a linker (which bridges the two ligands mentioned above via a chemical chain to form a ternary complex). PROTACs work by inducing ubiquitination tagging of POIs, which in turn are recognized and degraded by the 26S proteasome (Gu et al., 2018). PROTACs can directly remove target proteins, have catalytic and long-lasting effects, and can target traditional “undruggable” (undruggable) targets. Currently, PROTACs are mainly used to slow down the AD process indirectly by regulating Aβ metabolism, targeting Tau proteins, and removing misfolded proteins (Inuzuka et al., 2022). There have been PROTACs targeting Tau that have shown great potential in preclinical studies (Table 5).

**Table 5:** Experimental study of PROTACs in the treatment of AD.

Therapeutic drugs or methods	Experimental subjects	Model species	Key indicators of the AD model	Affected pathways/genes/proteins	References
C8	Mice, cells	HEK293, hTau-transgenic mice	Tau↓	-	Yao et al. (2024)
C004019	Mice, cells	HEK293, SH-SY5Y, hTau-transgenic mice, transgenic 3xTg-AD mice	Tau↓	-	Wang W. et al. (2021)
QC-01-175	Cells	Induced pluripotent stem cells	Tau↓	CRBN	Silva et al. (2019)
TH006	Mice	Transgenic 3xTg-AD mice	Tau↓	-	Chu et al. (2016)
Peptide1	Cells	SH-SY5Y	Tau↓	Keap1	Lu et al. (2018)

“↓” denotes reduced levels; “-” indicates that the article did not mention.

#### 4.5.1 Recruitment of VHL E3 ligase for tau degradation

In a more recent study, Yao et al. (2024) developed a compound, named C8, and conducted a series of explorations of its effects. It was found that C8 was able to reduce total Tau and phosphorylated Tau levels in HEK293-hTau cells, degrading Tau proteins in a time-dependent manner via UPS. In hTau overexpressing mice, C8 significantly reduced Tau protein levels in the hippocampal region of mice and improved cognitive function. In addition, it was shown that C8 has good blood–brain barrier permeability *in vivo*.

C004019, a small-molecule PROTAC with a molecular mass of 1035.29 daltons, was designed to recruit both Tau proteins and E3 ligase (Vhl), thereby selectively enhancing ubiquitination and proteasome-mediated protein hydrolysis of Tau proteins. Wang W. et al. (2021) conducted experiments in both in vivo and *in vitro* formats and showed that in vitro, C004019 significantly increased the level of ubiquitination of Tau in both HEK293-hTau cells and SH-SY5Y cells, and that this increase was eliminated by the proteasome inhibitor MG132. *In vivo* experiments, by perfusing C004019 into the intracerebroventricular compartment of 3xTg-AD mice, it was found that C004019 was able to reduce the levels of total Tau and phosphorylated Tau in the hippocampus and cortex, and improve the cognitive and synaptic functions of the mice.

#### 4.5.2 Recruitment of CRBN E3 ligase for tau degradation

Electron emission tomography (PET) technology is used to detect Tau protein deposition in the brain of AD patients in the clinic using specific PET probes that are able to bind to P-Tau in vivo in a conformation-dependent manner, such as the Tau PET probe 18F-T807. Researchers have utilized targeted protein degradation technology to convert 18F-T807 into the Tau degrader QC-01-175. Cereblon (CRBN) is the substrate receptor for the E3-ubiquitin ligase CRL4CRBN, which triggers the ubiquitination of Tau proteins and removes Tau proteins via the proteasomal degradation pathway. QC-01-175 recognizes Tau proteins and simultaneously binds CRBN to recruit the E3 ubiquitin ligase, forming a ternary complex used to mediate ubiquitination and proteasomal degradation of Tau protein. At the same time, QC-01-175 was able to restore Tau-mediated neuronal stress vulnerability induced by Aβ_(1–42)_ (Silva et al., 2019).

#### 4.5.3 Other E3 ligase recruitment strategies

Chu et al. (2016) designed and synthesized a series of multifunctional molecules that contain a part that recognizes Tau proteins, a part that binds to E3 ligase, and a part that penetrates cell membranes. Among these molecules, TH006 proved to be the most potent in inducing intracellular Tau protein degradation. Furthermore, TH006-mediated Tau degradation was able to reduce cytotoxicity induced by Aβ and modulate Tau levels in the brain in an AD mouse model.

Lu et al. (2018) designed and synthesized a PROTAC named Peptide 1 (Peptide 1). Keap1 is a substrate-adaptor protein of the Cullin3-dependent E3 ubiquitin ligase complex. Peptide 1 is able to enter cells and co-localize with intracellular Keap1, promoting the co-immunoprecipitation of Tau and Keap1, which in turn promotes Keap1-dependent Tau polyubiquitination and proteasome-dependent degradation to reduce Tau levels.

The aforementioned PROTACs play a beneficial role in targeting Tau protein degradation; however, PROTACs are large molecular proteins, and most of them still suffer from blood–brain barrier penetration challenges. In addition, the technical role of PROTACs requires the selection of E3 ligases that are highly expressed in the brain and have tissue specificity. In the future, new technologies will still be needed to overcome these challenges and thus promote the further development of PROTACs technology.

The evidence chain of most drugs that regulate UPS discussed in this paper is mainly based on animal and cell models, and is still in the preclinical stage as a whole. However, bortezomib and cilostazol have been approved for the treatment of cancer and intermittent claudication, respectively, and have shown good safety, which provides a favorable clinical basis for the treatment of AD. In addition, the clinical transformation of resveratrol provides important insights for us to understand the challenges of UPS regulators in the human body. A completed phase II clinical trial (nct01504854) showed that high-dose resveratrol showed acceptable safety in patients with AD and could significantly reduce the level of Aβ_40_ biomarker in cerebrospinal fluid (Moussa et al., 2017), but it failed to translate into significant improvement in cognitive function, which may be related to its relatively low permeability of the blood–brain barrier and complex multi-target characteristics.

## 5 Conclusion and Prospect

The dependent protein degradation pathway is an important way to maintain neuronal homeostasis. UPS is widely distributed in the human body and plays an important role in many physiological and pathological processes. Protein homeostasis is the core biological mechanism to maintain the dynamic balance of protein synthesis, folding, translocation, and degradation in cells. Its imbalance has been proven to be closely related to the pathogenesis of a variety of neurodegenerative diseases. In AD, the destruction of the protein steady-state network is considered to be the key factor driving the pathological cascade, which is directly involved in the deposition of Aβ and the formation of tau protein pathology. As the function of the protein quality control system, including UPS, declines with age, the clearance efficiency of abnormal proteins is further reduced, leading to the accumulation of Aβ and tau proteins. This paper discusses the process of ubiquitin modification and the role of UPS in AD, including the excessive accumulation of Aβ and tau proteins, the effect on neural cell functions such as synaptic plasticity, and UPS promoting the degradation of neurotoxic proteins in the brain to further restore the activity of damaged neurons. The process of ubiquitination modification is complex and diverse. This paper reviews the preclinical research progress of UPS in the treatment of AD in recent years, mainly focusing on the treatment of AD by regulating proteasome, ubiquitin ligase, and deubiquitinase. The treatment strategy for UPS has become one of the current research hotspots.

However, there are still major challenges in pushing UPS targeted therapy from the laboratory to the clinic. These challenges mainly include: ① Blood brain barrier penetration and bioavailability: many drugs face problems such as low oral bioavailability and difficulty in penetrating the blood–brain barrier. Future research should give priority to the optimization of drug structure and the development of new drug delivery systems (such as nanoparticles). ② Target specificity: Traditional Chinese medicine compounds have the characteristics of multi-channel and multi-target. In the process of targeting UPS, whether the components interact and whether they affect autophagy, inflammation, and other processes have brought difficulties to clarify its core mechanism of action and determine clinical biomarkers. In the future, imaging or body fluid markers that can accurately monitor UPS function should be developed, which is very important for the efficacy evaluation of traditional Chinese medicine in clinical trials. ③ Limitations of preclinical models: AD is the result of the interaction of multiple pathogenic factors. The current AD animal models can not fully simulate the complexity of human diseases, which affects the accuracy of preclinical data in predicting human curative effects to some extent. In this case, efforts should be made to build a multifactorial animal model combining gene technology, multi-omics technology, and artificial chips, so as to promote the transformation from laboratory to clinic.

The first generation monoclonal antibodies against Aβ monomer did not show a meaningful side in clinical trials, while the second generation monoclonal antibodies (such as aducanumab, lecanemab, donanemab) recently approved for clinical research clearly proved that targeting the Aβ pathway can delay the progression of early AD by directly clearing Aβ plaque in the brain (Kim et al., 2025). However, these drugs mainly act on extracellular Aβ and have limited ability to clear intracellular Aβ oligomers, phosphorylated tau, and other misfolded proteins. Interestingly, this is in sharp contrast to and potentially complementary to UPS regulators, suggesting that future treatment strategies can evolve from “single target” to “multi-target synergy.” For example, the combination of compounds that can activate proteasome or E3 ligase and anti-Aβ antibody can theoretically form the effect of “internal and external treatment”: the antibody is used to remove the plaque formed outside the cell, while UPS regulator is responsible for removing the Aβ oligomer and phosphorylated tau in the cell, so as to produce a synergistic effect, improve the efficacy and safety, and reduce the risk of possible side effects.

Based on the evidence sorted out in this review, among the numerous UPS modulators in preclinical research, some drugs show high short-term transformation potential. For example, cilostazol, a drug that has been approved for peripheral vascular disease and has clear safety, has been proven to enhance proteasome activity and reduce tau pathology, making it the most promising candidate drug for direct clinical validation of AD. Similarly, ursolic acid can directly activate the proteasome with a clear mechanism of action. It is an ideal compound for future drug development. In addition, E3 ligase modulators also show great potential to precisely regulate specific pathological pathways by upregulating Chip, Hrd1, or inhibiting Idol with small molecules. However, there are many E3 ligases, and only some of them have been found in the existing studies. Further exploration is still needed in the future in order to expand the targeted drug population of E3 ligases.

In conclusion, the treatment mode of ad in the future will change from “single target” to “multi-target synergy.” It will be the key to the next generation of therapy to explore the treatment method of intracellular and extracellular combined clearance of toxic proteins. Although there are many challenges in this process, with the in-depth exploration of the mechanism of UPS and the progress of targeted Technology (such as protacs), the treatment of AD by regulating the protein quality control system and restoring brain protein homeostasis is showing unprecedented broad prospects.
